# The use of online video consultations in the aftercare of orthopedic patients: a prospective case-control study

**DOI:** 10.1186/s12891-021-04653-3

**Published:** 2021-09-12

**Authors:** K Estel, G Weber, F Fellmer, L Richter, S Tsitsilonis, C Willy, DA Back

**Affiliations:** 1 Department for Traumatology and Orthopedics, Bundeswehr Hospital Berlin, Scharnhorststrasse 13, 10115 Berlin, Germany; 2 Medical Faculty, Charité - Universitätsmedizin Berlin, Charitéplatz 1, 10117 Berlin, Germany; 3 Center for Musculoskeletal Surgery, Charité - Universitätsmedizin Berlin, Augustenburger Platz 1, 13353 Berlin, Germany; 4 Dieter Scheffner Center for Medical Education and Educational Research, Charité – Universitätsmedizin Berlin, Charitéplatz 1, 10117 Berlin, Germany

**Keywords:** Video consultation, Orthopedics, Evaluation, Aftercare, Examination

## Abstract

**Background:**

Video consultations have proven to be an efficient source of support for patient-doctor interactions and have become increasingly used in orthopedics, especially during the COVID-19 pandemic. This study analyzed both patients’ and doctors’ acceptance of an orthopedic telemedical consultation (OTC) and compared the results of OTC examinations to the results of live consultation (LC) to identify discrepancies.

**Methods:**

The study was carried out in an orthopedic department of a German hospital between 2019 and 2020. After written informed consent was obtained, patients voluntarily presented for follow-up by OTC and LC. The experience with and attitudes toward OTC among both patients and doctors was evaluated (using Likert scale-scored and open questions, 26 to 28 items). The results of the OTC and LC examinations were compared using a 12-item checklist. The data were analyzed by quantitative and qualitative statistics.

**Results:**

A total of 53 patients were included, each of whom completed an OTC and an LC. The OTC was rated as pleasant, and the experience was rated as very satisfying (average rating on a 5-point Likert scale, with 1 indicating strong agreement: doctors: 1.2; patients: 1.3). Various technical and organizational challenges were identified. Compared to LC, OTC showed no significant differences in patient history or in inspection, palpation, or active range of motion results. Only for the functional or passive joint assessment did LC show significantly higher suitability (p < 0.05) than OTC. Recommendations for further procedures did not differ significantly between OTC and LC.

**Conclusions:**

Because of the high acceptance and the objective benefits of OTC and the similarity of clinical results with LC, OTC is recommendable for orthopedic follow-up examinations. To better assess joint functionality, meaningful digital alternatives for established examination methods should be further investigated.

**Supplementary Information:**

The online version contains supplementary material available at 10.1186/s12891-021-04653-3.

## Background

In the context of the current digital transformation in medicine, a broad spectrum of digital approaches are used, from electronic systems for managing patient data to device-based support for medical tasks and digital mobile service apps that provide “mobile health” services [1]. Some innovations are even referred to as disruptive technologies because of the lasting changes they have made to established offerings and processes [2].

Telemedicine is an established and broad term applied to digital tools. It involves the exchange of medical information between remote participants with the aim of creating the type of communication available in live consultations to improve the health of patients [3].

Experience with and the extent of established technical procedures vary among medical disciplines and geographic locations. For example, sending and receiving a remote diagnosis based on data sets alone has already been well-established procedure in medical specialties such as radiology [4]. In rural areas, experience with video-supported teleconsultation also occurred early [5]. In recent years, various changes, such as the constant improvement of internet speed, new legal frameworks and greater acceptance of digital solutions among people worldwide, have increased patients’ and healthcare providers’ attention to the topic of online video consultations as a reliable treatment option. Recently, contact restrictions during the COVID-19 pandemic in 2020 increased the relevance of online video consultations for doctor-patient contacts in many countries [6].

Telemedicine in orthopedics has already been used in various countries [5, 7, 8]. The first related studies at the end of the last century showed that telemedicine approaches could be used for outpatient orthopedic follow-up [5].

In recent years, many advantages of online consultations have been proven for orthopedics in particular and for other disciplines in general.

These positive aspects include providing consultations independent of the location, eliminating the need for patients to travel long distances to see their doctors and thus avoiding long absences from work [9], and reducing wait times [10]. Both insurance companies and health systems in general have reported reductions in costs [11, 12]. Overall, patient satisfaction is increased, e.g., with the use of online tools for postoperative care compared to “classical” personal aftercare concepts [9, 13]. The acceptance of digital contact methods was described as very positive by patients in some study results [6] but could not always be reproduced [14].

In addition to examining the satisfaction of patients and doctors with online video consultation, the present study aimed to investigate whether the examination results of patients with orthopedic diseases differed between online consultations and in-person consultation. Additionally, we wanted to gain insights into the quality of the consultations and their assessment by the patients and doctors involved.

## Materials and methods

### Study design and setting

**Fig. 1:**
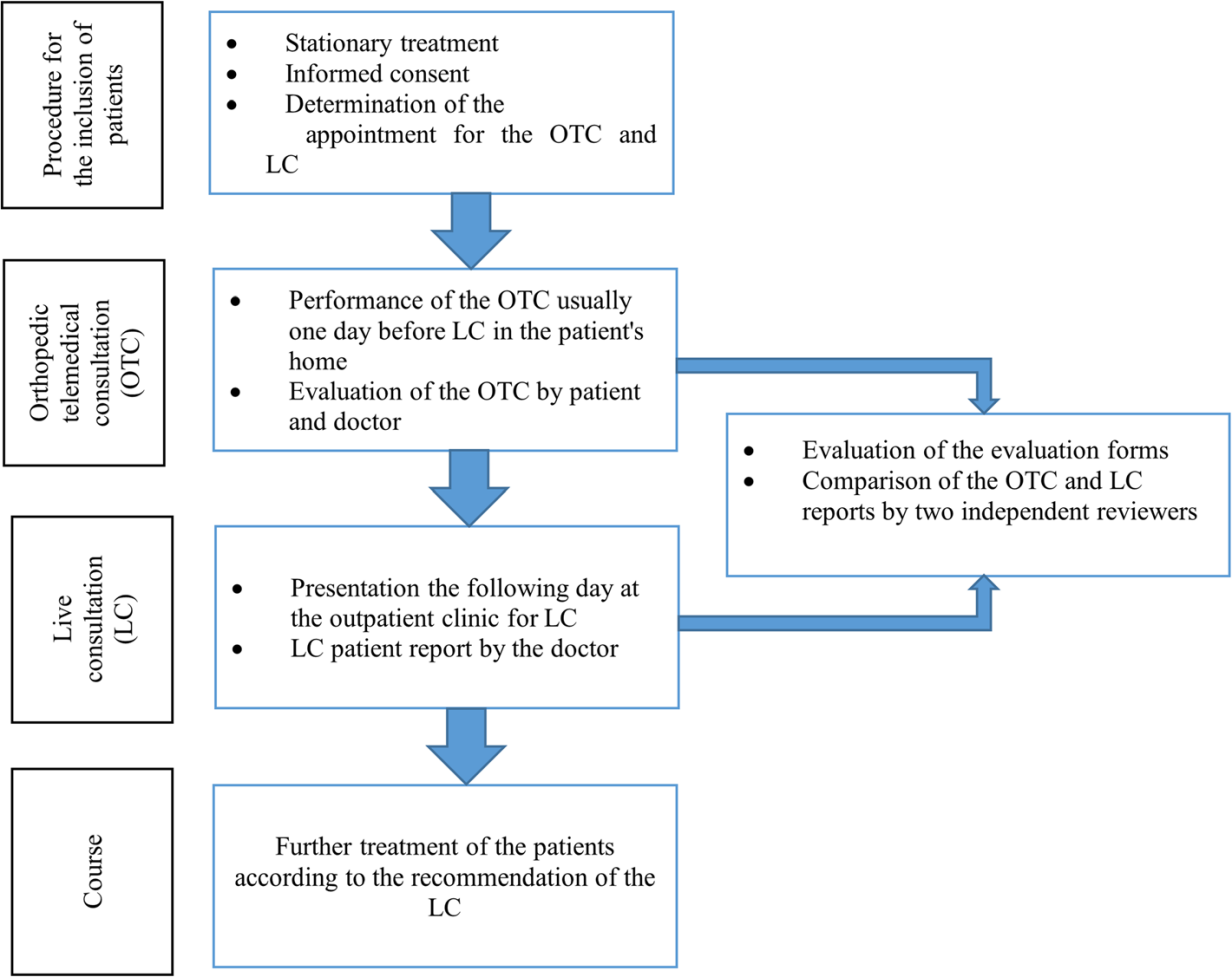
Overview of the methodological approach of the study

Before the live follow-up consultation was planned, patients of the Department Traumatology and Orthopedics, Bundeswehr Hospital Berlin, were offered an additional follow-up by means of online video consultation. Participation was voluntary. The patients gave their informed consent and had the option to withdraw from the study at any time without giving reasons and without consequences for their treatment. Participants who wished not to participate were asked their reasons for declining. The acquired data were pseudonymized. For both the online and live appointments, the results were documented in standardized reports and saved in the hospital information system (HIS). The concept of this case-control study included the plan that any determination for further therapy be reconfirmed during the in-person consultation. After the online appointment, the participating doctors were asked to assess their examination experience using an evaluation sheet, and the patients were asked to assess their consultation experience. Figure 1 gives an overview of the methodological approach used in the study. The examination findings were comparatively evaluated using a self-designed evaluation sheet. The research project was approved by the ethics committee of the Bundeswehr Hospital Berlin (Ärztekammer Berlin, No.: Eth-12/19). The study began in August 2019 and lasted until July 2020.

### Technical procedure

The real-time video conferences were conducted in accordance with applicable national law. End-to-end secure broadband connections between patients and doctors were established via a certified provider (Deutsche Arzt AG, Essen, Deutschland). Before the online appointment, the patients received a link and a password by email or SMS and were thus able to log into a protected waiting area, where they were called by the doctor. The connection was achievable regardless of the end device (mobile phone, tablet, etc.), operating system or manufacturer.

### Participation criteria

All participants in the study were soldiers in the German Federal Armed Forces, were over 18 years of age, had the technical ability to use Internet-supported computer communication and participated voluntarily in the study after receiving appropriate instruction.

Another prerequisite was a regular course of medical treatment. Exclusion criteria were the personal decision of the patients not to participate in the study and any other situation in which - in the opinion of the treating doctors - continued participation in the study would not be in the best interest of the patient.

### Structure of the doctor and patient questionnaires

The questionnaire for ***doctors*** (supplement 1) contained 25 questions (closed 5-point Likert scale-scored questions, partially open and open questions), which were subdivided into questions on demographic data (three questions), user preferences for digital services in the professional environment (five questions), general questions about the experience with the video consultation (punctuality, technical problems with making contact, atmosphere) (four questions), and specific questions about the experience of the video consultation (including sound/image quality, examination procedure, improvement options) (13 questions).

The questionnaire for ***patients*** (supplement 2) contained 27 questions (closed 5-point Likert scale-scored questions, partially open and open questions), which were divided into questions on demographic data (two questions), usage preferences for digital services in the medical sector (three questions), general questions about the experience with the video consultation (punctuality, technical problems with making contact, atmosphere) (four questions), specific questions about the experience with the video consultation (including sound/image quality, examination procedure, improvement options) (12 questions), and questions comparing the OTC to normal live consultations (including travel time, distance, burden of traveling to the hospital for the LC) (six questions).

Additionally, participants who did not wish to participate were verbally asked about their reasons for declining.

### Checklist of results on follow-up reports

Using an established checklist, two reviewers independently examined the examination reports from the online and personal consultations. This 12-item checklist included questions scored on a 5-point Likert scale, closed-selection options and free-answer questions (one question regarding patient history, five questions regarding findings, three questions regarding diagnostics, two questions regarding procedures and one open comment question). For patients who presented for wound management only, questions about range of movement and a functional examination were not applied.

### Statistics

The collected data were entered in a Microsoft Excel spreadsheet (Version 2016, Microsoft Inc., Redmond, WA, USA). For the questionnaire data, a quantitative data analysis and evaluation according to descriptive statistical methods was performed. For the analysis of the examination reports, we used IBM SPSS Statistics software (Version 23.0, IBM Corp., New York, USA). Cronbach’s alpha correlation coefficient was determined for the independent reviewers’ ratings to determine the objectivity of the evaluation. The Likert scale-scored data for the comparison of OTC and LC were comparatively analyzed with the Mann-Whitney U test. The free-text responses were examined by two authors to identify recurring statements using a systematic qualitative content analysis according to Mayring [15]: the free-text responses were selected from the questionnaires and examined for essential question content; a summary was performed to reduce the responses to a short text, and the summaries were analyzed; the results were interpreted; and a quality analysis was performed to ensure that the appropriate criteria were met.

## Results

Fifty-three patients (male: n = 51, female: n = 2) and six doctors (male: n = 4, female: n = 2) were included in this study. The average age of the participating patients was 36.4 (± 9.3) years. The average age of the doctors was 36.2 (± 2.6) years.

The clinical cases included 17 patients who had undergone previous shoulder surgery, 15 patients who had undergone knee surgery, 16 patients who required wound control after various surgeries, and five patients who had other clinical presentations (one patient after osteotomy for leg axis correction, one patient after humerus and lower leg fracture, one patient after total hip arthroplasty for coxarthrosis, and one patient each undergoing conservative treatment for knee joint or shoulder joint complaints). Patients with wounds presented immediately postoperatively, and joint examinations took place later, after the initial healing process (approximately 6 weeks, depending on the pathology). Two patients who were contacted refused to participate; one did not want to use any private devices from home, and the other patient had data protection concerns and unspecific discomfort about undergoing an online consultation. No participant was advised to discontinue the study.

## Doctors’ perceptions

**Fig. 2:**
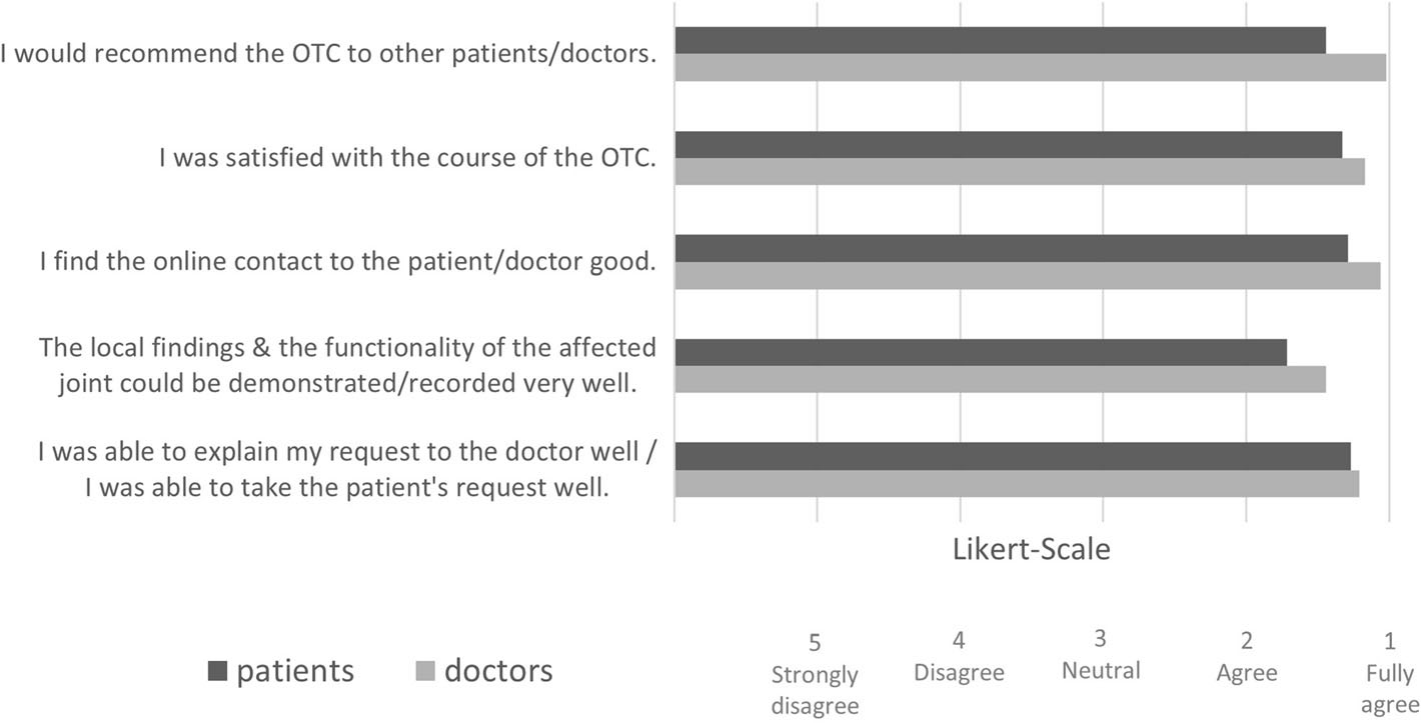
Evaluation of the experience with the OTC (*n* = 51) by patients and doctors (5-point Likert scale scoring)

Regarding the OTCs, the doctors evaluated almost all of the consultations as “very pleasant” (n = 48; 91 %). In 66 % (n = 35) of OTCs, no problems occurred from the doctors’ perspective. Problems (n = 18; 34 %) during OTCs that were indicated by the doctors were mainly of a technical nature (n = 15; 28 %). In one case, scheduling the appointment was cited as a problem, and in two cases, other problems were noted (poor Internet connection in the building, compatibility problems between the terminal device and the program). With regard to the transmission quality, in six cases, the doctors were less than satisfied with the sound, and in one case, the image was less than satisfactory (corresponding to a Likert scale rating of three out of five). Further evaluation results for the doctors’ OTC experience are shown in Fig. 2.

In a concluding open question on possible improvements to support the implementation of OTCs, the doctors primarily indicated optimizing organizational factors (n = 3) with fixed time slots for OTCs within the outpatient clinic’s medical consultation hours (n = 3) and the minimization of technical problems, specifically by ensuring the availability of stable Internet connections (n = 8).

## Evaluation of patients’ perceptions

98 % of all patients (n = 52) rated the atmosphere of the doctor-patient contact during OTC as positive (53 % (n = 28) as “immediately pleasant”, 45 % (n = 24) rated it as “unfamiliar at first, but then pleasant”. Only 2 % of the respondents found the atmosphere “aloof and persistently unsettling”. The patients reported no problems during the OTC in 62 % (n = 33) of the cases. The problems (n = 20; 38 %) during the OTC that were reported were mainly of a technical nature (n = 16; 80 %). In addition, in three cases, scheduling the appointment was cited as a problem, and in one case, access code transmission was problematic. In five cases, the patients were less than satisfied with the sound quality, and in one case, the image quality was less than satisfactory (corresponding to a Likert scale score of three; one response was missing). Further results regarding the evaluation of the patients’ OTC experiences are shown in Fig. 2. In the concluding open-ended question that asked about possible ways to improve the OTC, the patients primarily indicated the elimination of technical problems (n = 6), the use of image adjustment aids (e.g., selfie sticks) (n = 3), the optimization of scheduling appointments (n = 2), the provision of preparatory information about participating in an OTC (n = 2) and the development of a dedicated app (n = 1).

**Fig. 3:**
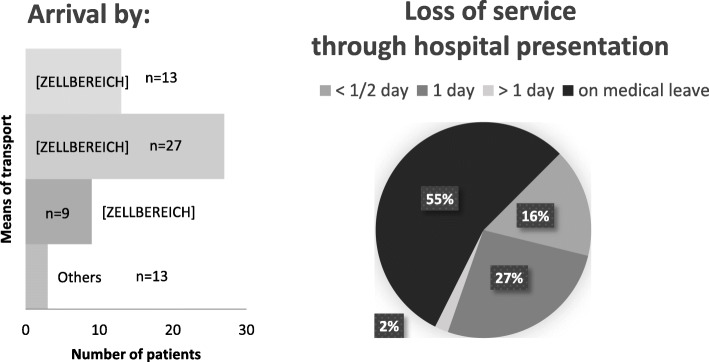
Mode of transport/arrival of the patients to the live consultation (n = 52) and their estimated loss of working time due to the hospital visit (n = 49, n = 4 abstentions)

Figure 3 presents an overview of the means of transportation the patients used to travel to the hospital and the work loss in days.

In addition, the average distance traveled between the point of stay and hospital was 170 km, with a maximum distance of 680 km. The average waiting time in the outpatient clinic was found to be 30.7 min (± 27.87, five missing answers) (compared to an almost punctual OTC). The patients felt slightly to moderately inconvenience by the need to present at the outpatient clinic (an average score of 4.2 on a scale of 0–10 (0 = not impaired at all; 10 = extremely impaired) (three answers were missing). 36 % of the interviewed patients reported physical complaints or pain while traveling to the hospital (three missing answers).

### Checklist for examination results

**Fig. 4:**
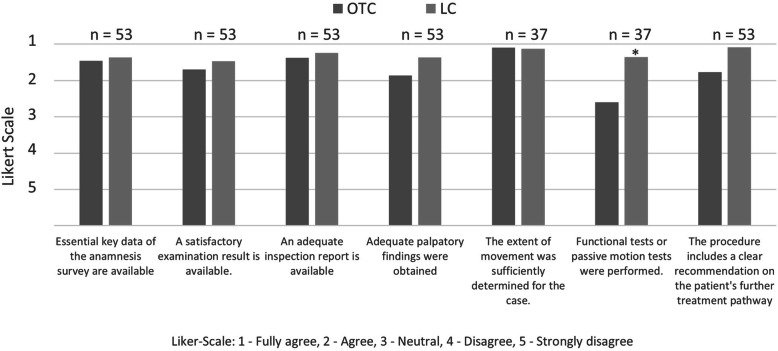
Comparison of ratings for the quality of the medical history, clinical examination and further treatment recommendations at the OTC and LC (* *p* = 0.031) (*n* = 51; range of motion and functional tests were not performed for patients undergoing check-ups for wounds only, n = 16; average scores)

The reports of the 53 included patients were evaluated, the results for different items were compared for the OTC and LC (Cronbach´s alpha for the independent reviewers: 0.802) (Fig. 4). Functional or passive motion tests were significantly more useful in the LC group than in the OTC group (*p* = 0.031).

In eight cases, radiographic imaging was conducted at the outpatient clinic during the live follow-up (three X-rays; one CT scan; two MRIs; two ultrasounds). Of these eight cases, four imaging examinations had been scheduled at a previous follow-up, two were recommendations at the OTC, and two were performed based on a new finding during the LC.

Only in two cases did the decisive core statements in the OTC recommendation for further therapy does not correspond with the LC. In one case, the need for the patient’s admission to the hospital for surgery was clear during the OTC, and the determination of the exact procedure to be performed was left until the live contact. In a second case, a different procedure was determined after X-ray control was performed at the LC.

## Discussion

In the context of the current digital transformation of the health care system and against the background of the COVID-19 pandemic and its need for reduced physical contact, telemedicine, with its potential to reduce wait times for patients or to allow postoperative control, is becoming increasingly important, including in orthopedics [5, 7, 8]. Because it requires a complete restructuring of existing processes in some areas, telemedicine may even be viewed as a disruptive technology [9].

The presented case-control study examined the benefits of OTC among patients and doctors in the setting of orthopedic follow-up examinations. In particular, its technical feasibility, doctors’ assessments of its usefulness and possible obstacles and challenges were analyzed. In addition, the results of the OTC and LC were compared for each patient to provide initial indications regarding the quality of OTC.

One noteworthy result of the study was the positive evaluation of the OTC experience by patients and doctors. A clearly positive attitude toward recommending OTC to other patients or doctors was ascertainable, and at the same time, the atmosphere during the OTC was subjectively perceived as pleasant. Therefore, a previously reported negative effect of online consultations alone, without in-person interaction, on the doctor-patient relationship could not be confirmed [14]. A randomized controlled trial showed that orthopedic patients were satisfied with the inclusion of OTCs within their treatment [16]. One reason for the positive evaluation of OTC could be the familiar surroundings, which the patients indicated as an advantage and which is confirmed by the existing literature [17]. However, the potential elimination of long travel and wait times also contributed to the patients’ positive evaluations of the OTCs. This is consistent with study results of other authors [9, 10, 18].

In the present study, OTC was mainly used for surgical aftercare. The literature has already shown that online consultations are practicable for this purpose [19, 20]. Nevertheless, it must be stated that not all patients agree to online consultations and that online consultations should be regarded as integrated support for medical treatment pathways [10]. When relevant disease symptoms that are red flags are detected in an online consultation, an LC should take place immediately, and if necessary, treatment should be initiated. In this way, OTC could help to focus LCs on prediagnosed patients and those who have started therapy, to the benefit of all involved patients.

In most cases, the OTCs took place without technical problems. However, approximately one-third of the OTC presented noticeable challenges, a proportion that is comparable to the findings of other studies [21, 22]. In the course of this study, low bandwidth in rural areas in Germany was found to impair consultations, a problem that has been described for other regions worldwide [19, 22]. These problems require major attention as they may lower the acceptance of telemedicine and the willingness to use it in the future [23]. A strong and stable internet connection should thus be regarded as crucial and essential [19].

In addition to the good acceptance and the objective benefits of the technology, the study also found comparatively good quality of the physician-patient consultation between LCs and OTCs. Different studies have suggested that the physical examination of patients with musculoskeletal diseases via telemedicine is very limited [24–26]. However, in the present study, the quality of medical history and inspection were nearly equivalent between LCs and OTCs. Similar results have also been demonstrated for the other aspects of physical examinations, such as palpation and active range of motion. It has already been demonstrated that examinations of the hip, knee, shoulder, and elbow joint are feasible using telemedicine [24–26]. The examination quality could be increased by giving patients a checklist containing preparatory information in advance of the telemedicine consultation [27]. Although OTC demonstrated limitations in the present study in terms of functional tests of joints using classic examination methods, it seems that with modifications of those functional tests and the use of assistive devices, it is possible to make reliable statements about this important component of the examination [27, 28]. Most importantly, analyses of the established procedures for each case individually showed that the OTC recommendations for further treatment were similar to the live consultation recommendations in 96 % of the cases. While the design and evidence level of this study was not strong enough to allow clinical recommendations to be formed – thus emphasizing the need for further randomized and controlled trials (RCTs) – the results tend to suggest that decisions about the further course of therapeutic actions in orthopedics can be made on the basis of OTC.

Additionally, in this study, we analyzed the distance from the patients’ residences to our clinic, the different forms of transportation used, and the time required for LC that would have otherwise been spent on work commitments. We found that very long travel distances often had to be covered by the patients themselves. The fact that travel time and waiting times at the outpatient clinic were eliminated when OTC was used suggests that must shorter time commitments were required, thus making more of the patients’ time available for work [9, 10]. However, care must be taken to ensure that enough time is allowed for doctor-patient contact in OTC settings. Furthermore, when OTC is used, patients who are in pain are not affected by travel to the clinic or by painful experiences that may result from traveling. In addition to the patients themselves, other people (e.g., accompanying drivers) are impacted by the need to travel for in-person health care visits. Additionally, almost 50 % of the patients needed to miss work for at least half a day, unless they were already on sick leave. These factors indicate a significant strain on human and financial resources that could be reduced by implementing OTC [11, 12, 29].

The study has some limitations. The direct comparison of the OTC with live consultations cannot guarantee the same objectivity as an RCT. Additionally, the voluntary nature of participation could include a bias in the participants’ basic attitudes toward OTC and thus could make the evaluation data appear falsely positive. Furthermore, it must be critically noted that there was no recording of the duration of the online and live consultations, which would have provided an important additional measure of effectiveness. Finally, the number of selected patients was not large and comprehensive enough to make statements about the application of OTC for all orthopedic disease entities. The voluntary nature of participation could also have led to biased selection. Participants who were already interested in digital topics might have been more willing to participate in studies on this topic. In the future, more patients with a wider range of ages and a more balanced gender distribution should be included.

## Conclusions

Based on the findings of this study, it seems advisable to establish OTCs at orthopedic clinics since they provide good options for the follow-up of orthopedic patients. In addition, they offer advantages for treating doctors and patients, such as saving time, avoiding long journeys to the clinic, and perhaps even reducing material and personnel costs. The limitations in terms of orthopedic examinations suggest the need for digital adaptations of functional tests. Technical problems need to be identified and analyzed to ensure that they are minimized during the daily clinical routine.

## Supplementary Information

**Additional file 1:**

**Additional file 2:**

## Data Availability

The datasets used and/or analyzed during the current study are available from the corresponding author upon reasonable request.

## Code availability

Not applicable for that section.
