# Supplementary material for: The use of online video consultations in the aftercare of orthopedic patients: a prospective case-control study
Source: BMC Musculoskelet Disord. 2021 Sep 12;22:774. doi: 10.1186/s12891-021-04653-3 (PMC8435151; doi:10.1186/s12891-021-04653-3)
Supplement: Supplementary file 1 — Additional file 1: [file 12891_2021_4653_MOESM1_ESM.docx]

Supplement 1 - questionnaire for ***doctors*** *presented in the paper*

What is your age? ___ years

Sex: ⬜ female ⬜ male

How would you rate the contact with the patient and the atmosphere compared to face-to-face contact?

⬜ Immediately pleasant - from the beginning there was no difference to a personal contact.

⬜ First unfamiliar at first, but pleasant as it progressed and similar to personal contact.

⬜ Aloof and permanently unsetting.... (was not able to express myself...).

⬜ Personally I found the contact unpleasant and impersonal.

What were the problems in the current appointment of online video consultation?

⬜ Technical problems (please specify):___________

⬜ Finding/keeping appointments

⬜ Problems explaining my concerns/complaints.

⬜ Others (please specify): ____________________

⬜There were no problems.

|  | **Further questions regarding the use of online video consultation** | Fully agree | Agree | Neutral | Disagree | Strongly disagree |
| --- | --- | --- | --- | --- | --- | --- |
|  | 1. I was able to take the patient´s request well. | ⬜ | ⬜ | ⬜ | ⬜ | ⬜ |
|  | 1. The local findings of the affected joint could be recorded well. | ⬜ | ⬜ | ⬜ | ⬜ | ⬜ |
|  | 1. The functionality of the affected joint could be recorded well. | ⬜ | ⬜ | ⬜ | ⬜ | ⬜ |
|  | 1. As a physician, I think it is good to be able to contact the patient via OTC. | ⬜ | ⬜ | ⬜ | ⬜ | ⬜ |
|  | 1. I was satisfied with the course of the OTC. | ⬜ | ⬜ | ⬜ | ⬜ | ⬜ |
|  | 1. I would recommend the OTC to other doctors. | ⬜ | ⬜ | ⬜ | ⬜ | ⬜ |
|  | 1. What can be improved for the implementation of an online video consultation? (free answer) ______________________________________________________________________________________________________________________________   ______________________________________________________________________________________________________________________________ | | | | | |
|  | 1. What differences did you experience (advantages/disadvantages) between online vs. „live“ consultations (free answer)?   ______________________________________________________________________________________________________________________________________________________________________________________________________________ | | | | | |
